# Occipital Encephalocele with Multiple Birth Defects: A Case Report

**DOI:** 10.31729/jnma.6542

**Published:** 2021-10-31

**Authors:** Bikash Pyakurel, Anita Lamichhane, Bikash Bhandari, Navachandra Oli, Somraj Lamichhane

**Affiliations:** 1 Department of Pediatrics, Lumblnl Medical College, Palpa, Nepal; 2 Department of Neurosurgery, Lumblnl Medical College, Palpa, Nepal

**Keywords:** *birth defects*, *cleft lip*, *cleft palate*, *encephalocele*

## Abstract

A full-term female baby presented at 24 hours of life at the emergency department with occipital encephalocele, bilateral cleft lip, and cleft palate. She was born to a second gravida mother with no consanguinity between the parents. On examination, encephalocele was 10centimeters x 7centimeters in size with bilateral cleft lip and palate. It presents the opportunity for healthcare professionals to learn about a group of congenital neurological disorders in the content of a rare case presentation and highlights the importance of ultrasonography in the antenatal period for the detection of neural tube defects in the early stage for proper counselling and management. A compulsory prenatal diagnosis of the suspected family should be done by the intervention of the public sector of any country so that we can prevent and avoid abnormal birth.

## INTRODUCTION

Encephalocele is a congenital malformation characterized by a protrusion of the brain tissue and/or meninges through a skull defect; classified into primary which is present at birth while secondary encephalocele is acquired and commonly due to trauma or post-surgical defect.^1^ Myelomeningocele, meningocele, encephalocele, and anencephaly comprise 80% of all Neural tube defects (NTDs). Encephaloceles represent 15%-20% of all NTDs. The incidence of congenital encephalocele is estimated at 1 in 10,000 live births.^2^ The incidence is still high in developing countries.^3,4^ The prevalence of selected NTDs is 4.0 (95 % Confidence Interval= 2.0-7.0) per 10,000 children in Nepal.^5^

## CASE REPORT

**Figure 1:**
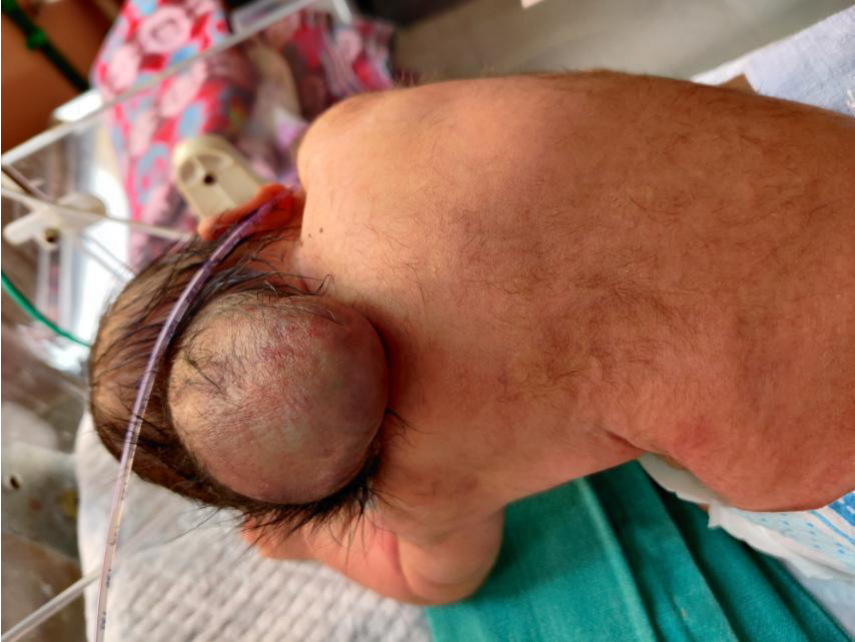
Showing occipital encephalocele about 7cm x 10cm in size.

**Figure 2:**
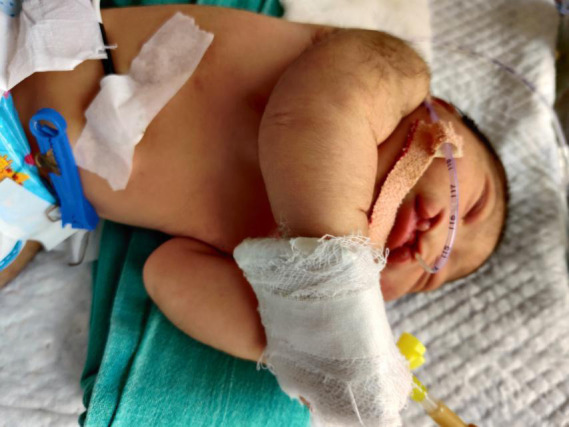
Showing bilateral cleft lip and palate of the neonate.

A female baby presented at 24hrs of life at the emergency department of Lumbini Medical College and Teaching Hospital Palpa, Nepal after a spontaneous vaginal delivery at home. She was born full-term with a birth weight of 2.8kgs to a 27years old second gravida mother with one live baby at home. Birth events were uneventful; the baby cried immediately after birth. Upon arrival to hospital, paediatrician on the call noted a bulging occipital mass with 10cm x 7cm in size (Figure 1) with bilateral cleft lip and palate (Figure 2).

The baby was then shifted to the Neonatal Intensive Care Unit (NICU) where she was kept under an overhead warmer in the left lateral position and was kept nil per oral. Nasogastric feeding was started as she had difficulty in sucking due to the cleft lip and palate. Detailed examination revealed an active baby moving all four limbs normally with no neurological deficit. Head circumference was 28.5cm (<5^th^ percentile), Anterior fontanel was open and at the level. There was no murmur, the baby had passed meconium within 24hours after birth. There were two umbilical arteries and one umbilical vein. Limbs were normal. Regarding the antenatal history, the mother attended her Antenatal Care visits two times with ultrasonography (USG) done twice without any significant anomalies in USG. She did not take any iron, calcium, or folic acid supplementation during pregnancy. Her past obstetrical history was insignificant without any diseases or medication history during pregnancy. Her other child is healthy with no evidential defect. There was no history of consanguinity among the parents.

**Figure 3:**
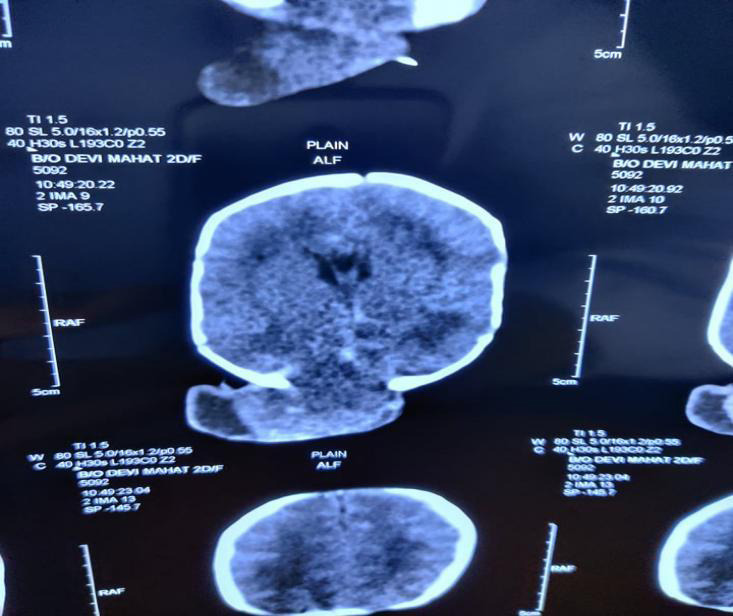
CT SCAN brain showing a bony defect of approximately 3*4cm over the occipital region with herniation of meninges along with brain parenchyma (occipital and cerebellar tissue).

The neurosurgical team was consulted and they recommended Computerized Tomography (CT) scan (Figure 3) which showed a bony defect approximately 3x4cm over the occipital region with herniation of meninges and brain parenchyma (occipital and cerebral tissue).

A provisional diagnosis of Encephalocele with Bilateral cleft lip and palate was made. The neurosurgeon advised interval excision of encephalocele along with calvarium reconstruction. The parents were counselled but they did not give the consent immediately. Instead, the subject was discharged on request from the hospital assuring us that the parents would return soon after arranging the personal work at home. They were taught spoon-feeding as the special spoon for the cleft palate was not available in our area. But we lost the patient on follow-up.

## DISCUSSION

Occipital encephalocele presents as a mass in the occipital region usually covered by skin. They are the most common type of encephalocele. It is more frequent in females than males.^5^ They are often associated with other midline anomalies such as hypertelorism, broad nasal root, cleft lip, and cleft palate.^6^ This is similar to our case reported showing occipital encephalocele with cleft lip and palate. Other associations include microcephaly, microphthalmia, cleft lip and palate, polydactyly, polycystic kidneys, and ambiguous genitalia.^7^ Occipital encephaloceles occur due to a defect in the fusion of occipital bone. The occipital bone develops from two sources. The failure of fusion of these two parts of occipital bone has resulted in the defect in this case. Such midline defects are associated with other midline lesions.^8^ This fetus presented with bilateral cleft lip and cleft palate. Such variable association of midline defects with occipital encephalocele can be attributed to multifactorial aetiology. More than 80% of encephalocele cases are not associated with certain genetic or chromosomal abnormalities.^9^ The incidence is between 1 in 3000 to 1 in 10,000 live births; approximately 90% of them involve the midline. Magnetic resonance imaging is the method of choice in diagnosis and surgery is the best option for the treatment of Occipital Encephalocele. The prognosis of patients born with occipital encephalocele depends on the size of the defect and the amount of brain tissue herniated into the encephalocele. Overall morbidity and mortality are high despite advanced surgical management but have been significantly improved in recent years thanks to sophisticated high-resolution imaging, adequate and proper surgical treatment, and decent post-operative care.^10^ Factors that determine the prognosis of patients diagnosed with occipital encephaloceles include the size of the sac, the contents of the neural tissue, the presence of hydrocephalus, infections, and pathologies that accompany the condition.^11^

The aim of surgery is resection of the sac, maximum preservation of herniated brain tissue, and closure of the defect, and surgery should be performed as early as possible. In a technical note mentioned by Bozinov, et al. for the surgical closure and reconstruction of a large occipital encephalocele without parenchymal excision, initially, the cystic portion is removed so that a partial reduction of the encephalocele is achieved. Six months later, the surgical closure of the defect is performed, with preservation of the occipital and cerebellar parenchyma, by incising the tentorium and retracting the cortex to the newly created infratentorial space. The bony defect is covered with autologous osseous graft harvested from parietal bone and reconstructed.^12^ In some patients with gliotic tissue can be completely excised without postoperative neurological deficit. Whereas, Occipital Encephalocele associated with hydrocephalus needs a ventriculoperitoneal shunting.^11^ But with associated anomalies, the patients' prognosis becomes rather poor. Hence there is a need for early prenatal diagnosis of such congenital defects. Limited literature regarding the aetiology and risk factors associated with occipital encephaloceles warrants additional prospective studies with larger populations. There is a need for the development of a good surveillance program with a full-proof reporting system. In fact, in the surveillance manual for congenital anomalies developed by WHO, ICBDSR, and CDC encephalocele, cleft lip and cleft palate have been included not only because of their ease of diagnosis but also because there is a potential for prevention, early diagnosis, and treatment.^13^
